# Identification of three new ‘*Candidatus* Liberibacter solanacearum’ haplotypes in four psyllid species (Hemiptera: Psylloidea)

**DOI:** 10.1038/s41598-022-24032-9

**Published:** 2022-11-30

**Authors:** Kylie D. Swisher Grimm, David R. Horton, Tamera M. Lewis, Stephen F. Garczynski, Andrew S. Jensen, Brian A. Charlton

**Affiliations:** 1 Temperate Tree Fruit and Vegetable Research Unit, USDA-ARS, 24106 N. Bunn Road, Prosser, WA 99350 USA; 2 Temperate Tree Fruit and Vegetable Research Unit, USDA-ARS, 5230 Konnowac Pass Road, Wapato, WA 98951 USA; 3 Northwest Potato Research Consortium, Olathe, CO USA; 4 Klamath Basin Research and Extension Center, Oregon State University, 6941 Washburn Way, Klamath Falls, OR 97603 USA

**Keywords:** Molecular biology, Entomology, Bacteria

## Abstract

Eleven haplotypes of the bacterium, *‘Candidatus* Liberibacter solanacearum’, have been identified worldwide, several of which infect important agricultural crops. In the United States, haplotypes A and B are associated with yield and quality losses in potato, tomato, and other crops of the Solanaceae. Both haplotypes are vectored by potato psyllid, *Bactericera cockerelli*. Recently, a third haplotype, designated F, was identified in southern Oregon potato fields. To identify the vector of this haplotype, psyllids of multiple species were collected from yellow sticky cards placed near potato fields during two growing seasons. Over 2700 specimens were tested for *‘Ca.* L. solanacearum’ by polymerase chain reaction. Forty-seven psyllids harbored the bacterium. The infected specimens comprised four psyllid species in two families, Aphalaridae and Triozidae (Hemiptera: Psylloidea). Nucleic acid and/or amino acid sequence analysis of the ‘*Ca.* L. solanacearum’ 16S ribosomal RNA, 50S ribosomal proteins L10/L12, and outer membrane protein identified three new haplotypes of the bacterium, designated as Aph1, Aph2 and Aph3, including two variants of Aph2 (Aph2a and Aph2b). The impact of these new haplotypes on solanaceous or other crops is not known. The vector of *‘Ca.* L. solanacearum’ haplotype F was not detected in this study.

## Introduction

Agricultural crops around the globe suffer from quality and yield losses caused by the phloem-limited bacterium, ‘*Candidatus* Liberibacter solanacearum’. Eleven haplotypes of ‘*Ca.* L. solanacearum’ have been described, many of which show different biological traits, causing disease symptoms in specific crops in geographically distinct regions around the world. Despite the evidence of biological function of specific haplotypes, classification of these genetic variants as biotypes has yet to be made by the research community. Insect associates of the haplotypes have invariably been species of psyllids (Hemiptera: Psylloidea) also present in these geographically distinct regions.

‘*Ca.* L. solanacearum’ haplotypes A, B, and F occur in solanaceous crops and weeds. These haplotypes are associated with disease symptoms known as ‘permanente del tomate’ in tomato (Mexico) and ‘zebra chip’ in potato (globally). Aboveground symptoms include yellowing or purpling of foliage, shortened internodes and premature senescence^1,2^. Tomatoes and peppers from infected plants are small and deformed, while tubers from infected potato plants have prominent striped patterns that make them unmarketable. Haplotype A has been found in North America, Central America, South America, and New Zealand, while haplotype B has been found in North America and Central America and appears to induce more severe disease symptoms than haplotype A^1,3,4^. ‘*Ca.* L. solanacearum’ haplotypes A and B are both vectored by the potato psyllid, *Bactericera cockerelli* (Šulc) (Hemiptera: Triozidae). Haplotype B has also been detected in a relative of *B. cockerelli*, the triozid *Bactericera maculipennis* (Crawford)^5,6^. This species develops on Convolvulaceae. It is not yet known whether Convolvulaceae is a host of ‘*Ca.* L. solanacearum’. ‘*Ca.* L. solanacearum’ haplotype F has been found only in potato and only in southern Oregon, United States. The vector of haplotype F has not been reported^2^. Recently, a fourth haplotype associated with Solanaceae, ‘*Ca.* L. solanacearum’ haplotype G, has been isolated from decades-old herbarium samples of *Solanum umbelliferum* from southern California. The new haplotype is most closely related to haplotype F based on genetic analyses^7^. Haplotype G was also recently found in present day *S. umbelliferum* and potato psyllids were found in association with this plant species, indicating that the vector of haplotype G likely is *B. cockerelli*^8^*.*

‘*Ca.* L. solanacearum’ haplotypes C, D, and E are associated with disease symptoms in apiaceous crops such as carrot, celery, and parsnip^9–11^. These haplotypes induce proliferation of roots or stems, curling of stems and leaves, and purpling or yellowing of foliage. Haplotype C is found in northern Europe, while haplotypes D and E are found in the Mediterranean region and northern Africa^1,12^. ‘*Ca.* L. solanacearum’ haplotype C is transmitted by the carrot psyllid, *Trioza apicalis* (Foerster), and has also been found in *Trioza anthrisci* Burckhardt (Triozidae)^9,13–15^. Haplotypes D and E are both transmitted by *Bactericera trigonica* (Hodkinson) (Triozidae), and haplotype E appears to be acquired but not transmitted by a second triozid species, *Bactericera tremblayi* (Wagner)^16–18^. A fourth haplotype, ‘*Ca.* L. solanacearum’ haplotype H, was recently identified in symptomatic carrot and parsnip plants collected in Finland^19^. Symptoms included leaf purpling and curling. Weedy species of Polygonaceae showing red discoloration of leaves were also found to harbor ‘*Ca.* L. solanacearum’ haplotype H in this same region. The insect vector of haplotype H is unknown^19^.

‘*Ca.* L. solanacearum’ haplotype U was identified in stinging nettle (*Urtica dioica*; Urticaceae) and in a nettle-specialized psyllid (*Trioza urticae* (L.)) at locations in Finland^15^. Symptoms of ‘*Ca.* L. solanacearum’ haplotype U infection are yellowing or purpling of leaf margins. Genetic analysis of haplotype U identified it as most closely related to haplotypes A and D, despite the geographical distances between the regions where these haplotypes have been found.

Finally, a survey to identify psyllid and ‘*Ca.* L. solanacearum’ populations in carrot in Scotland recently identified two additional haplotypes of ‘*Ca.* L. solanacearum’ designated as Cras1 and Cras2 due to their presence in species of *Craspedolepta* psyllids (Aphalaridae)^20^. These haplotypes are genetically most closely related to haplotype H. Neither Cras1 nor Cras 2 has yet been detected in plants, though it is suspected that they will be found in a non-crop host plant of the *Craspedolepta* psyllids, such as *Chamaenerion angustifolium* (Onagraceae)^20^.

While knowledge of the ‘*Ca.* L. solanacearum’ pathogen continues to expand with the identification of novel haplotypes, only haplotypes A, B, C, D, and E have been subjected to in-depth analyses to explore their relationship with their respective psyllid vectors and host plants, as well as their economic effects on crops. In an effort to identify the psyllid vector of the newly identified ‘*Ca.* L. solanacearum’ haplotype F found in the Klamath Basin of Oregon, psyllid specimens were collected from sticky card traps located in or near potato fields in 2018 and 2019 and tested for ‘*Ca.* L. solanacearum.’ The potato psyllid, *B. cockerelli*, is found only at very low levels in this region (Charlton, personal observation), suggesting that psyllids other than potato psyllid may transmit this pathogen to potato. Since there is currently no method to control ‘*Ca.* L. solanacearum’ itself, identification and characterization of insect vectors could allow growers to target the vector of the pathogen for control of disease. ‘*Ca.* L. solanacearum’-infected psyllids were identified by targeting the cytochrome oxidase I gene and confirming identifications with standard morphological tools and consultation with the taxonomic literature. This included taxonomic examination of specimens collected using sweep nets from the same region that provided the sticky card specimens, identifying the specimens using standard taxonomic tools, and verifying molecular match with the sticky card specimens. ‘*Ca.* L. solanacearum’ haplotypes were identified by targeting the 16S ribosomal RNA, 50S ribosomal protein subunit L10/L12, and the outer member membrane protein genes. These analyses led to the identification of novel ‘*Ca.* L. solanacearum haplotypes in four non-potato psyllid species. Haplotype F was not found in any psyllid specimens.

## Results

### Detection of ‘*Ca.* L. solanacearum’

A total of 1,226 and 1,525 psyllids were removed from yellow sticky cards placed in or near potato fields in Klamath Basin during the 2018 and 2019 field seasons, respectively. All samples were tested for the presence of *‘Ca.* L. solanacearum’ by PCR targeting the 16S rRNA gene. In 2018, 25 psyllids tested positive for the bacterium with an infection rate of 2.04% and in 2019, 22 psyllids tested positive with an infection rate of 1.44%.

Identification of the ‘*Ca.* L. solanacearum’ present in each infected sample was verified through targeting of three standard genes, including the 16S rRNA gene, 50S ribosomal subunit protein L10 (rplJ) and L12 (rplL) genes, and the outer membrane protein gene. For analysis of the 16S rRNA gene, only 23 and 13 of the total samples from 2018 and 2019, respectively, resulted in sequences of high quality. Consensus sequences were used in BLAST searches to confirm that these target sequences were ‘*Ca.* L. solanacearum.’ All sequence profiles were identified with homology to ‘*Ca.* L. solanacearum,’ with the highest nucleotide identity ranging from 99.81–99.91%. To confirm homology with ‘*Ca.* L. solanacearum,’ individual BLAST searches were also performed against other Liberibacter species, including *‘Ca.* L. africanus’ (97.84–98.22% nucleic acid identity), ‘*Ca.* L. americanus’ (94.69–94.89% identity), ‘*Ca.* L. asiaticus (97.16–97.41% identity), ‘*Ca.* L. brunswickensis (97.41–97.66% identity), ‘*Ca.* L. ctenarytaina’ (94.16–94.55% identity), and ‘*Ca.* L. europaeus’ (94.39–94.84% identity).

For analysis of the 50S ribosomal subunit protein L10 (rplJ) and L12 (rplL) genes, only 12 and 18 of the total samples from 2018 and 2019, respectively, resulted in sequences of high quality. BLAST results identified homology to ‘*Ca.* L. solanacearum,’ with the highest nucleotide identity ranging from 98.22–99.69%. To confirm homology with ‘*Ca.* L. solanacearum,’ individual BLAST searches were also performed against other Liberibacter species, including *‘Ca.* L. africanus’ (76.72–77.66% nucleic acid identity), ‘*Ca.* L. americanus’ (67.52–69.15% identity), ‘*Ca.* L. asiaticus (76.89–77.56% identity), and ‘*Ca.* L. ctenarytaina’ (74.52–75.61% identity). For analysis of the outer membrane protein gene, only 14 and 18 of the total samples from 2018 and 2019, respectively, resulted in sequences of high quality. A BLAST search of the consensus sequences identified homology to *‘Ca.* L. solanacearum,’ with the highest nucleotide identity ranging from 97.05–97.93%. To confirm homology with ‘*Ca.* L. solanacearum,’ individual BLAST searches were also performed against other Liberibacter species, including *‘Ca.* L. africanus’ (67.85–69.03% nucleic acid identity), ‘*Ca.* L. americanus’ (69.73–70.99% identity), and ‘*Ca.* L. asiaticus (69.59–70.51% identity).

### Analysis of ‘*Ca.* L. solanacearum’ haplotypes

**Figure 1:**
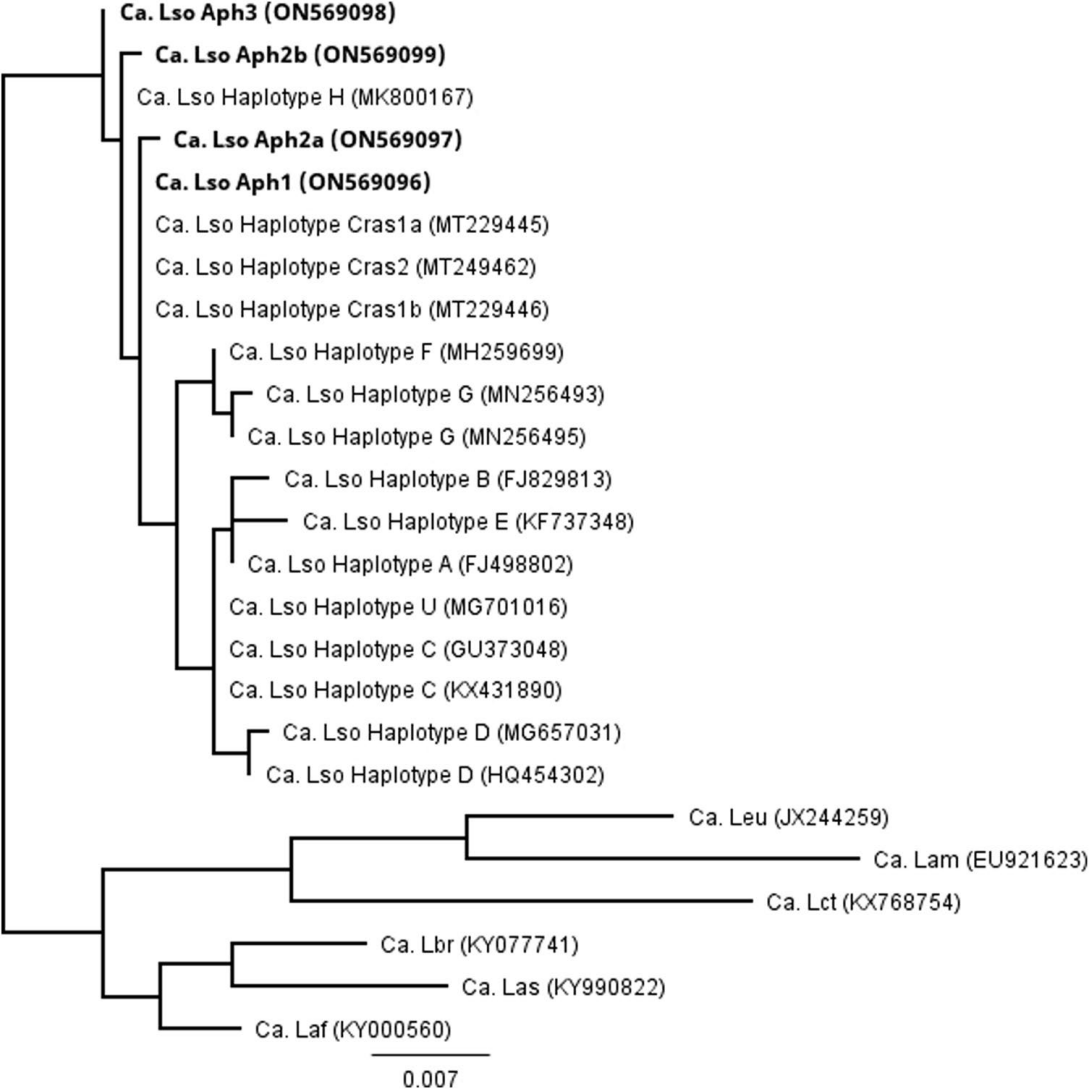
Unrooted phylogenetic tree constructed using a 1,023-basepair region of the 16S ribosomal RNA gene from *Ca.* Liberibacter species. Abbreviations used were Ca. Lso (*Ca.* L. solanacearum) Ca. Laf (*Ca.* L. africanus), Ca. Lam (*Ca.* L. americanus), Ca. Las (*Ca.* L. asiaticus), Ca. Lbr (*Ca.* L. brunswickensis), Ca. Lct (*Ca.* L. ctenarytaina), and Ca. Leu (*Ca.* L. europaeus). GenBank accession numbers are indicated in parentheses and specimens identified in this study are indicated in bold font. Bar length represents substitutions per site.

**Figure 2:**
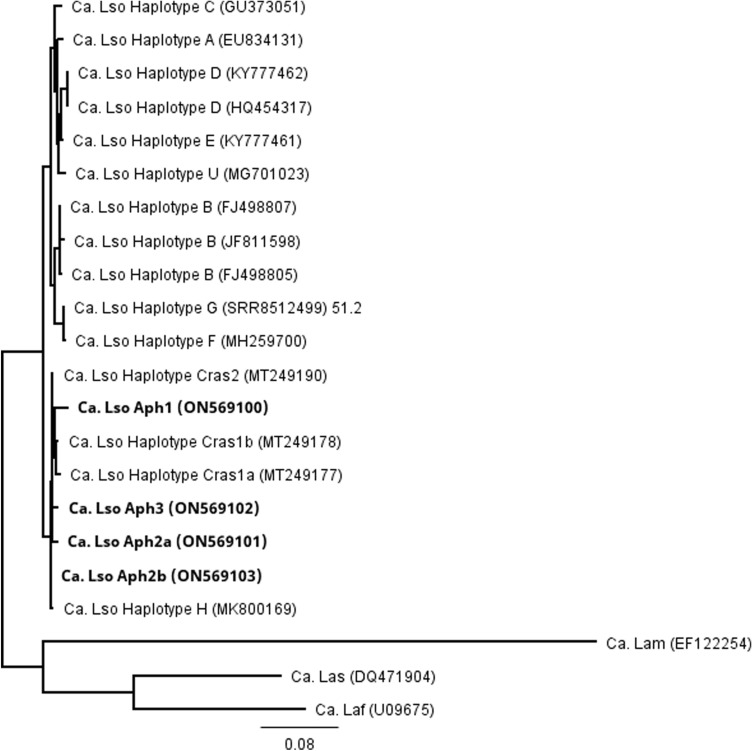
Unrooted phylogenetic tree constructed using a 626-basepair region of the 50S ribosomal proteins L10 and L12 genes from *Ca.* Liberibacter species. Abbreviations used were Ca. Lso (*Ca.* L. solanacearum) Ca. Laf (*Ca.* L. africanus), Ca. Lam (*Ca.* L. americanus), and Ca. Las (*Ca.* L. asiaticus). GenBank accession numbers are indicated in parentheses and specimens identified in this study are indicated in bold font. Bar length represents substitutions per site.

**Figure 3:**
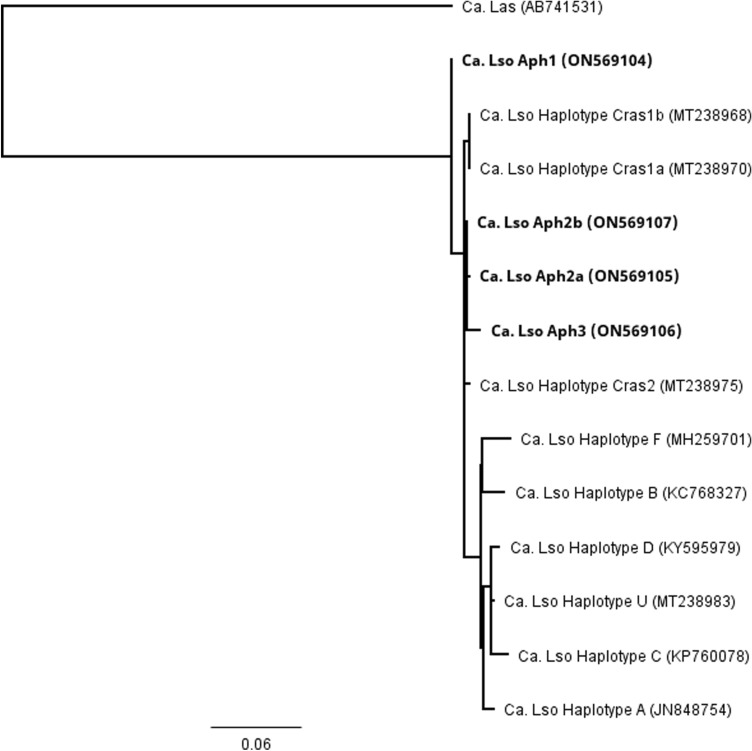
Unrooted phylogenetic tree constructed using a 586-basepair region of the outer membrane protein gene from *Ca.* Liberibacter species. Abbreviations used were Ca. Lso (*Ca.* L. solanacearum) and Ca. Las (*Ca.* L. asiaticus). GenBank accession numbers are indicated in parentheses and specimens identified in this study are indicated in bold font. Bar length represents substitutions per site.

Multiple sequence alignments of the cloned and direct-sequenced products were performed for the 16S rRNA sequences generated from 36 individual samples, the 50S ribosomal protein gene sequences generated from 30 individual samples, and the outer membrane protein sequences generated from 32 individual samples. Four unique sequence profiles were identified for each gene of interest. A second multiple sequence alignment was performed for these four unique sequences and ‘*Ca.* Liberibacter’ species available in GenBank for the 16S rRNA, 50S ribosomal protein, and outer membrane protein genes. Generation of a phylogenetic tree for the 16S rRNA gene identified the four novel sequence profiles as closely associated with ‘*Ca.* L. solanacearum’ haplotypes H, Cras1a, Cras1b, and Cras2 (Fig. 1). Similarly, a phylogenetic tree was generated for the 50S ribosomal protein genes and identified the new sequence profiles as closely related to ‘*Ca.* L. solanacearum’ haplotypes H, Cras1a, Cras1b, and Cras2 (Fig. 2). A phylogenetic tree generated for the outer membrane protein gene identified the new haplotypes as closely related to ‘*Ca.* L. solanacearum’ haplotypes Cras1a, Cras1b, and Cras2 (Fig. 3).

The 16S rRNA gene sequence for the four new profiles were aligned with the previously described, unique ‘*Ca.* L. solanacearum’ haplotypes, including haplotype A, B, C, D, E, F, G, H, U, Cras1a, Cras1b and Cras2 (Table 1). All four new sequences had distinct nucleotide differences. Three of the four new sequences contained single nucleotide polymorphisms (SNPs) that were unique and were not present in any other known haplotype. The four novel sequences were initially designated here New1, New2, New3, and New4 (GenBank ON569096, ON569097, ON569098, and ON569099, respectively).

**Table 1:**
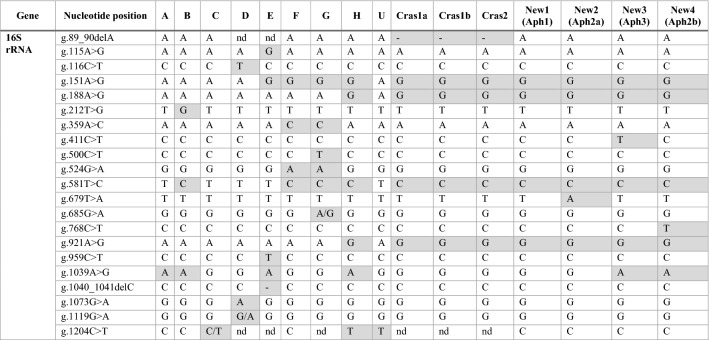
Single nucleotide polymorphism comparison in the 16S rRNA gene between ‘*Ca.* L. solanacearum’ haplotypes A, B, C, D, E, F, G, H, U, Cras1a, Cras1b, Cras2, and the four new sequence profiles described herein, New1 (ON569096; haplotype Aph1), New2 (ON569097; haplotype Aph2a), New 3(ON569098; haplotype Aph3), and New4 (ON569099; haplotype Aph2b).

The 50S ribosomal protein gene sequences for the four new profiles were aligned with the previously described, unique ‘*Ca.* L. solanacearum’ haplotypes, including haplotype A, B, C, D, E, F, G, H, U, Cras1a, Cras1b, and Cras2 (Table 2). Each of the four new sequences had distinct nucleotide differences and were designated here as New1, New2, New3, and New4 (GenBank ON569100, ON569101, ON569102, and ON569103, respectively). New1, New2, and New3 contained seven, two, and three SNPs, respectively, that were unique and not present in any other known haplotype. New3 also contained a nucleotide deletion that was not present in any other known haplotype. These sequences were translated to the amino acid sequence for the 50S ribosomal protein subunit L10 and L12, and percent identity matrices were generated following the multiple sequence alignment of a 71 amino acid region of the 50S ribosomal protein subunit L10 and a 104 amino acid region of the 50S ribosomal protein subunit L12. For L10, the sequences for New2, New3 and New4 were 100% identical, and these sequences were 100% identical to ‘*Ca.* L. solanacearum’ haplotypes H, Cras1b and Cras2. For L12, the sequences for New2 and New4 were 100% identical, and these sequences were 100% identical to ‘*Ca.* L. solanacearum’ haplotypes H and Cras2.

**Table 2:**
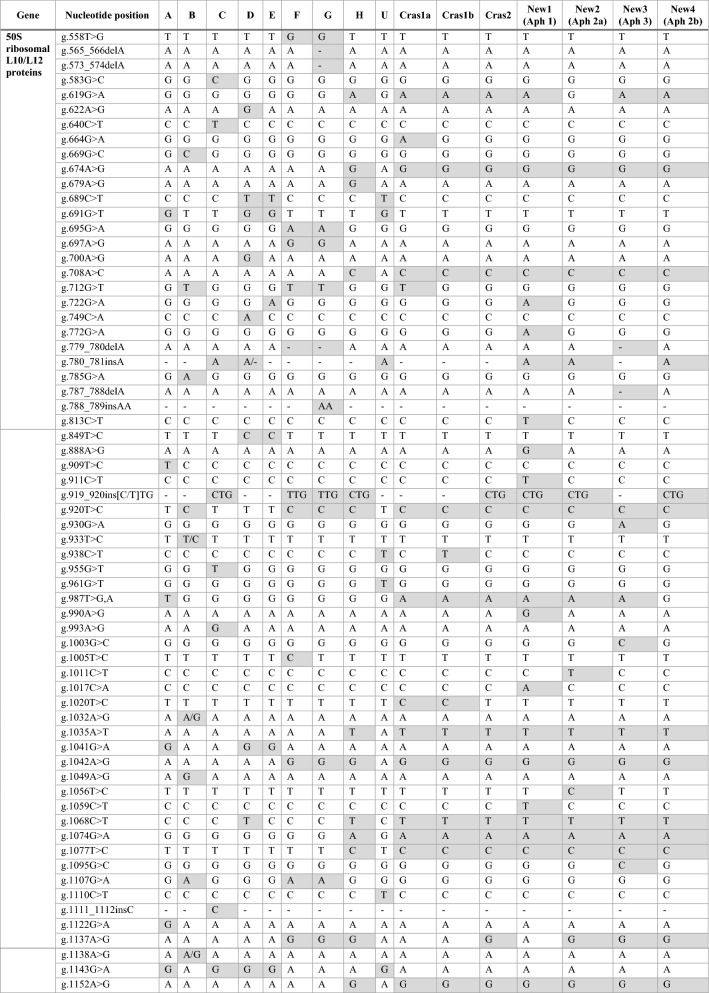
Single nucleotide polymorphism comparison in the 50S ribosomal L10 and L12 protein genes between ‘*Ca.* L. solanacearum’ haplotypes A, B, C, D, E, F, G, H, U, Cras1a, Cras1b, Cras2, and the four new sequence profiles, New1 (ON569100; haplotype Aph1), New2 (ON569101; haplotype Aph2a), New3 (ON569102; haplotype Aph3), and New4 (ON569103; haplotype Aph2b).

The outer membrane protein gene sequence for each of the four new profiles were also aligned with the previously described, unique ‘*Ca.* L. solanacearum’ haplotypes, including A, B, C, D, F, U, Cras1, and Cras2 (Table 3). Again, each of the four new sequences had distinct nucleotide differences and were designated here as New1, New2, New3, and New4 (GenBank ON569104, ON569105, ON569106, and ON569107, respectively). New1, New2, and New3 had four, one, and five SNPs, respectively, that were unique and not present in any other known haplotype. A percent identity matrix was generated following the multiple sequence alignment of a 191 amino acid region and New2 and New4 were 100% identical. These sequences were not identical to any other ‘*Ca.* L. solanacearum’ haplotype.

**Table 3:**
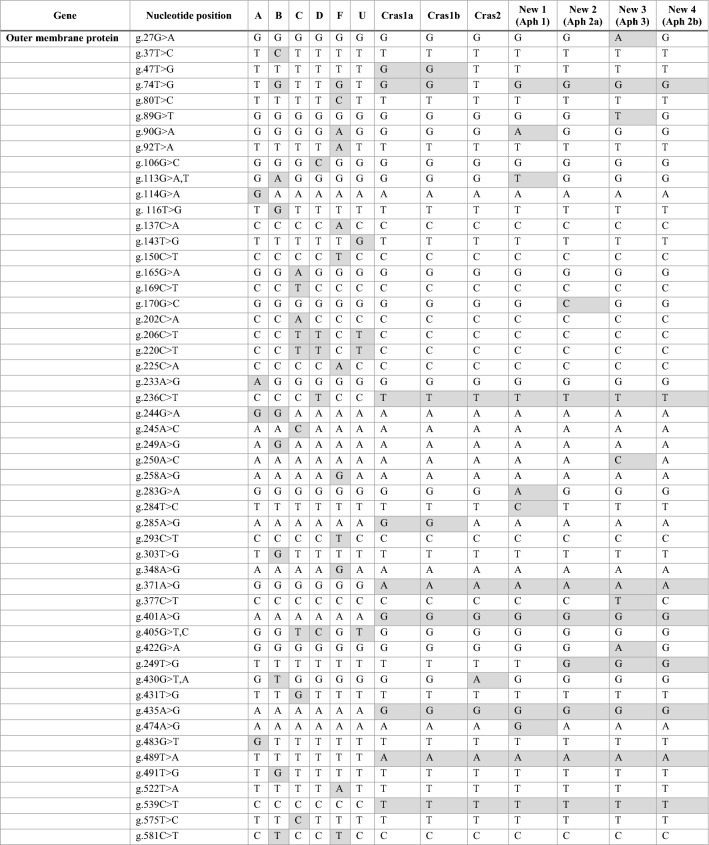
Single nucleotide polymorphism comparison in the outer membrane protein gene between ‘*Ca.* L. solanacearum’ haplotypes A, B, C, D, F, U, Cras1a, Cras1b, Cras2, and the four new sequence profiles, New1 (ON569104; haplotype Aph1), New2 (ON569105; haplotype Aph2a), New3 (ON569106; haplotype Aph3), and New4 (ON569107; haplotype Aph2b).

### Identification of psyllid species carrying ‘*Ca.* L. solanacearum’

CO1 sequences were identical in 27 of 46 psyllid specimens found to be carrying ‘*Ca.* L. solanacearum’ (GenBank Accession ON569108; Table 4). BLAST analysis identified homology of the psyllids to an unidentified Aphalarinae species (GenBank Accession KR582021). Two additional specimens shared a SNP within the CO1 gene when compared to these 27 specimens (GenBank Accession ON569109). Twenty-six of the twenty-nine specimens were found to harbor the *‘Ca.* L. solanacearum’ haplotype New1, while the haplotype in the other three specimens was undetermined due to poor sequence quality (Table 4). Near identical CO1 sequences were generated for four other specimens, with two specimens having a SNP at different locations. BLAST analysis of the three consensus sequences (GenBank Accessions ON569110, ON569111, and ON569112) identified homology to an unidentified species of *Craspedolepta* (GenBank Accession KR578677). The identification as *Craspedolepta* appears to be a misidentification of *Aphalara* (see Discussion). Two specimens harbored *‘Ca.* L. solanacearum’ New2, one was infected with New4, and one infection could not be identified due to poor sequence quality (Table 4). For nine other psyllid specimens, the CO1 sequences were nearly identical, with four specimens containing a single SNP from the others (GenBank Accessions ON569113 and ON569114). Interestingly, three clones of the CO1 gene from one specimen were sequenced, and one clone contained this same SNP, suggesting that the SNP could be the result of amplification or sequencing errors (GenBank Accessions ON569115 and ON569116). A BLAST analysis of both consensus sequences identified homology to *Craspedolepta* species (GenBank Accession MG401317). Again, the identification as *Craspedolepta* appears to be a misidentification of *Aphalara*. The nine specimens harbored *‘Ca.* L. solanacearum’ New1 (n = 2), New2 (n = 4), or New3 (n = 3) (Table 4). The remaining four *‘Ca.* L. solanacearum’-infected psyllid specimens produced identical CO1 sequences (GenBank Accession ON569117), and BLAST analysis of the consensus sequence identified homology to *Heterotrioza chenopodii* (GenBank Accession MT021799). The four specimens were infected with the *‘Ca.* L. solanacearum’ New2 based on 16S rRNA sequencing alone (Table 4).

**Table 4:** Species of psyllids identified through molecular and morphological analysis and the associated ‘*Ca.* L. solanacearum’ haplotypes identified in this study. *Aphalara* species identifications are with varying levels of certainty.

Group	CO1 Accessions	Associated species	No. of specimens	Associated sequence profiles & ‘*Ca.* L. solanacearum’ haplotypes
1	ON569108 and ON569109	*A. loca*	29	26 = New1 (Aph1) 3 = undetermined
2	ON569110, ON569111 and ON569112	*A. persicaria*	4	2 = New2 (Aph2a) 1 = New4 (Aph2b) 1 = undetermined
3	ON569113, ON569114, and clones ON569115/ ON569116	*A. curta*	9	2 = New1 (Aph1) 4 = New2 (Aph2a) 3 = New3 (Aph3)
4	ON569117	*H. chenopodii*	4	4 = New2 (Aph2a)

**Figure 4:**
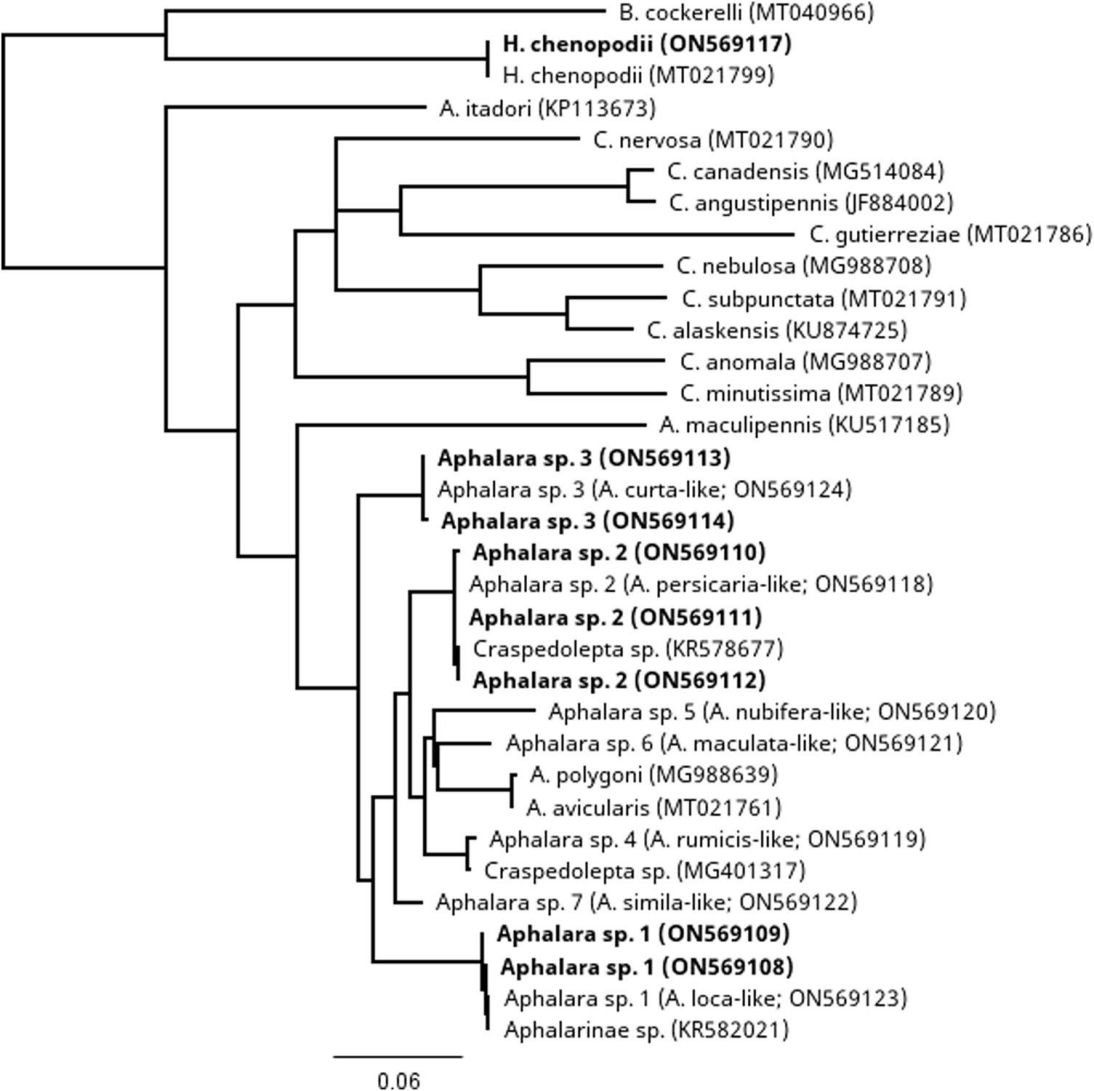
Unrooted phylogenetic tree constructed using a region of the mitochondrial cytochrome oxidase I gene ranging from 441- to 709-basepairs from *Aphalara* species, *Craspedolepta* species, *Heterotrioza chenopodii*, *Bactericera cockerelli,* the unknown specimens used in this study, and the specimens identified in the taxonomic study from collections made in the Pacific Northwest U.S. GenBank accession numbers are indicated in parentheses and ‘*Ca.* L. solanacearum’-infected specimens from Klamath Basin are indicated in bold font. Bar length represents substitutions per site.

Generation of a phylogenetic tree (Fig. 4) indicated that ‘*Ca.* L. solanacearum’-infected specimens from Klamath Basin (shown as bold font in Fig. 4) grouped with supplemental specimens that we assigned by morphology to formally named species (see Supplemental Table S1). The infected specimens grouped with three of our morphologically identified species: *Aphalara* sp. 1 (= *Aphalara loca*; GenBank Accession ON569123), *Aphalara* sp. 2 (*Aphalara persicaria*; ON569118), and *Aphalara* sp. 3 (*Aphalara curta*; ON569124). None of the other four species identified morphologically (*Aphalara rumicis*, *A. nubifera*, *A. maculata*, and *A. simila*; Supplemental Table S1) grouped molecularly with specimens found to harbor ‘*Ca.* L. solanacearum’ (Fig. 4). We caution that species identifications are of varying certainty due either to minor departures in morphology from described species or because described species most closely matching our specimens had not previously been collected from the study region (Supplemental Table S1). Identification of *Aphalara* sp. 1 as *A. loca* seems to have had the best support among the seven morphospecies (Supplemental Table S1). All supplementary specimens keyed morphologically to *Aphalara*, with no *Craspedolepta* detected in the samples. ‘*Ca.* L. solanacearum’-positive specimens and supplementary specimens also grouped together in the tree separately from the *Craspedolepta* clade (Fig. 4). Lastly, the identification of *H. chenopodii* specimens (harboring ‘*Ca.* L. solanacearum’ New2) in our collection from Klamath Basin was confirmed molecularly (Fig. 4).

## Discussion

A small subset of psyllid specimens collected from sticky cards (2.04 and 1.44% in 2018 and 2019, respectively) were infected with ‘*Ca.* L. solanacearum’. Sequencing and phylogenetic analyses were done to identify *‘Ca.* L. solanacearum’ haplotypes, and to identify each infected psyllid specimen.

Targeting of the 16S ribosomal RNA gene, the 50S ribosomal proteins L10/L12 genes, and the outer membrane protein gene identified the samples as most closely related to *‘Ca.* L. solanacearum,’ rather than the other Liberibacter species. Further, the sequences from each of these genes did not belong to any known *‘Ca.* L. solanacearum’ haplotype, but instead were identified as four novel ‘*Ca.* L. solanacearum’ nucleic acid sequences (New1, New2, New3, and New4). Phylogenetic comparison of the nucleic acid sequences from each of these genes identified these new sequences as most closely related to the newly described *Ca.* L. solanacearum haplotype H, identified in Finland from plant species in the Polygonaceae, and haplotypes Cras1a/1b and Cras2, discovered in Scotland infecting *Craspedolepta* (Aphalaridae) psyllids^19,20^. ‘*Ca.* L. solanacearum’ haplotype F was not identified.

Subsequent analysis of the amino acid sequences for the 50S ribosomal proteins and the outer membrane protein identified only three unique sequences from the four novel nucleic acid sequences. Because the 16S ribosomal RNA sequence was unique for both the New1 and New3 sequences, and the amino acid sequences were unique for 3 or 2 of the protein sequences analyzed, respectively, these samples are designated here as novel *‘Ca.* L. solanacearum’ haplotypes Aph1 and Aph3. While New2 and New4 had different nucleic acid sequences for the 16S ribosomal RNA gene, the identical amino acid sequences for the three proteins targeted (50S L10 and L12, and the outer membrane protein) suggest that these samples are genetic variants of the same haplotype, designated here as Aph2a and Aph2b.

The *‘Ca.* L. solanacearum’ haplotypes Cras1a/1b and Cras2 were named based on their identification in *Craspedolepta* psyllids, in an effort to move away from the confusing alphabetic designation of haplotypes^20^. Attempts to identify infected psyllids in this study were not immediately successful due to quality of the specimens on the sticky cards. Difficulties were magnified because of the taxonomic complexity of *Aphalara* and its close relative *Craspedolepta*^21,22^. Morphological and molecular examination of supplemental specimens of *Aphalara* collected from the Northwestern U.S. confirmed identification of infected psyllids as belonging to *Aphalara*, although assigning specimens to formally named species was somewhat uncertain depending upon species (Supplemental Table S1): *Aphalara loca* Caldwell (Aph1 haplotype), *Aphalara persicaria* Caldwell (Aph2a and Aph2b haplotypes), and *Aphalara curta* Caldwell (Aph1, Aph2a, and Aph3 haplotypes). No infected *Craspedolepta* were detected on cards. Specimens identified as *Craspedolepta* sp. in GenBank (KR578677 and MG401317) clustered with *Aphalara* molecularly rather than with either Old or New World *Craspedolepta* (Fig. 4). The two genera are closely related and are separated in keys by sometimes subtle differences in characteristics of structures on the head and by host plant (mostly Asteraceae for *Craspedolepta*, mostly Polygonaceae for *Aphalara*). Mistakes in identifying the two genera are not unexpected, and the two *Craspedolepta* sp. sequences in GenBank appear to be from *Aphalara* and not *Craspedolepta*.

Identification of specimens as *Aphalara* led to the selection of “Aph” for haplotype designation. We are most confident about our identification of *A. loca* (*Aphalara* sp. 1), which also was the species represented by the largest number of infected specimens on traps (Table 5). Additional sequencing data of the CO1 gene from specimens negative for ‘*Ca.* L. solanacearum’ found that *A. loca* was overrepresented in the infection group relative to its numerical presence on cards (Table 5). The host plant of this psyllid in the study region appears to be the weedy Old World species *Polygonum aviculare* (Supplemental Table S1), but whether this plant harbors ‘*Ca.* L. solanacearum’ haplotype Aph1 has yet to be determined. We were somewhat less certain of the other *Aphalara* identifications. *Aphalara persicaria* (*Aphalara* sp. 2), in which was found the Aph2a and 2b haplotypes of ‘*Ca.* L. solanacearum’, is reported to have a species of Polygonaceae (*Persicaria lapathifolia*) as its host plant in the Eastern U.S.^22^. This plant species is present in the Pacific Northwest, but whether it also hosts *A. persicaria* in the Pacific Northwest or is a host of the Aph2a and 2b ‘*Ca.* L. solanacearum’ haplotypes has yet to be determined. *Persicaria lapathifolia* hosts haplotype H of ‘*Ca.* L. solanacearum’ in Finland^19^, and consequently would seem to merit attention as a possible host for ‘*Ca.* L. solanacearum’ in North America. *Aphalara* sp. 3, tentatively identified as *A. curta*, was found to harbor Aph1, Aph2a, and Aph3 (Table 4). The host plant of *A. curta* is unknown but probably is a species of Polygonaceae. In addition to its presence in two species of *Aphalara*, we also detected the Aph2a haplotype of ‘*Ca.* L. solanacearum’ in four specimens of a completely unrelated psyllid, the triozid *Heterotrioza chenopodii*. Additional sequencing data of the CO1 gene from specimens negative for ‘*Ca.* L. solanacearum’ found that *A. curta* was underrepresented in the infection group relative to its numerical presence on cards (Table 5). *A. loca, A. persicaria*, and *A. curta* made up the highest proportion of psyllids recovered from the sticky cards, while *A. simila* and *A. rumicis* were very uncommon (Table 5). Almost no other psyllid taxa were recovered from the sticky cards.

**Table 5:** Taxonomic composition generated from sequencing the CO1 gene from a subset of the specimens removed from the sticky card traps and percentage of infected specimens for each species.

Species	Taxonomic composition on cards (as % of total psyllids); n = 355	Percentage infected
*Aphalara curta*	43.1	19.6
*Aphalara loca*	24.8	63.0
*Aphalara persicaria*	16.6	8.7
*Heterotrioza chenopodii*	7.0	8.7
*Aphalara nubifera*	4.5	0
*Aphalara maculata*	23	0
*Aphalara simila*	< 1	0
*Aphalara rumicis*	< 1	0
Other	< 1	0

Host plants of *Aphalara* are primarily species of Polygonaceae, although some species develop on Brassicaceae, Ranunculaceae, Caryophyllaceae, or Primulaceae^21,23,24^. *Heterotrioza chenopodii* develops on Amaranthaceae such as species of *Atriplex*^25^. In the Klamath Basin, Polygonaceae is common in wetland areas (*Polygonum*) and in pastures (*Rumex*)^26^, while *Atriplex* is found across the region in southern Oregon^25^. Despite having very different host plants, two species of *Aphalara* and the triozid *H. chenopodii* were all found to harbor ‘*Ca.* L. solanacearum’ haplotype Aph2a. This finding suggests that these three psyllids feed on at least one plant species that is also host to the ‘*Ca.* L. solanacearum’ haplotype Aph2a. While psyllid species tend to be specialized in developmental hosts, they are quite generalized in their feeding habits and will settle on and ingest from many different plant species as they disperse through the landscape^27^. Indeed, wintering *Aphalara loca—*the species found extensively to be infected by ‘*Ca.* L. solanacearum’ in this study—has been shown to harbor plant DNA from multiple plant families, ingested apparently while dispersing from its annual host, *Polygonum aviculare*, as the plants died down in late summer and autumn^6^. All of the infected psyllids analyzed in this study were collected from yellow sticky traps placed in or near potato fields in the Klamath Basin. It seems likely that plant species serving as reservoirs of these new ‘*Ca.* L. solanacearum’ haplotypes may also be present in or near these potato fields.

Our study raises an important point about the challenges faced in understanding epidemiology of previously unknown haplotypes of ‘*Ca.* Liberibacter solanacearum’ caused by uncertainties in identifying the psyllid host. Challenges in identifying psyllid species may complicate efforts to predict presence of new Liberibacter haplotypes in commercial crops or in non-crop hosts. The Psylloidea currently consists of ~ 4,000 known species with perhaps as many species as yet undescribed^28^. Many geographic faunas are poorly studied, including the North American complex of *Aphalara*^22^. *Aphalara* is a genus of over 40 species distributed primarily in the Holarctic region^22,24^. Host plants have yet to be identified for a number of species in this genus, including at least 2 species collected in this study (Supplemental Table S1). *Aphalara* species are difficult to separate from one another because morphological differences can be subtle and because important traits vary geographically within species^22^. Much of the information for the North American fauna of *Aphalara* comes from a single publication^29^, which sometimes failed to include important diagnostic characters, and sometimes based new descriptions on a limited number of specimens^22^. Thirteen species of *Aphalara* are currently listed for North America^21,22^, although this total likely underestimates actual diversity^30^. These uncertainties contributed to difficulties in assigning formal taxonomic names to *Aphalara* specimens collected in this study (Supplemental Table S1) and indicates that considerable effort is still needed to improve the overall taxonomy of this psyllid genus.

Finally, despite attempts by our group for several years, no plant species have yet been identified harboring any of these new ‘*Ca.* L. solanacearum’ haplotypes. Future research is needed to determine if these novel haplotypes have any effect on potatoes or other crops in the Klamath Basin region of Oregon and California, or elsewhere. A previous study on ‘*Ca.* L. solanacearum’ haplotype C has indicated that this haplotype can infect potato in Finland but does not produce any symptoms of zebra chip disease despite causing proliferation of roots, stem/root distortion and aboveground symptoms of purpling, yellowing and leaf curl in apiaceous crops^9,10,31^. Thus, it is possible that the ‘*Ca.* L. solanacearum’ haplotypes newly identified herein do not cause yield or quality losses in potato or other crops. If this is true, it could largely be because potato is not a true host of the Aphilaridae and Triozidae species identified from sticky traps in this study and would only be used as an intermittent food source. However, further research is needed for this to be determined.

## Materials and methods

### Insect samples

Yellow sticky cards (4 × 6 inch; Alpha Scents, Inc, Canby, OR) were placed in or near potato fields in the Klamath Basin of Oregon and California between June 10th and September 10th in 2018 and 2019 to monitor psyllid specimens in the potato growing region^32,33^. Each sticky card was placed in the field for the duration of one week, upon which sticky cards were collected and replaced for the following week. This process was repeated over the duration of the summer. Psyllids were removed from yellow sticky cards using a pipette tip and placed into a sterile 1.5 ml Eppendorf tube for nucleic acid extraction.

### Nucleic acid extractions and PCR analysis

Nucleic acids were extracted from each individual psyllid specimen using a cetyl trimethyl ammonium bromide extraction procedure^34^. All samples were resuspended in 50 µl of purified H_2_O for further analysis.

All psyllid samples were analyzed for the presence of *‘Ca.* L. solanacearum’ using standard polymerase chain reaction (PCR) with primers OA2/OI2c^35,36^ that target the 16S rRNA gene and Go Taq DNA polymerase (Promega, Madison, WI) using the conditions outlined in^2^. Any sample that produced a band of the expected size (1168-basepairs) was considered positive for ‘*Ca.* L. solanacearum’, regardless of the brightness of the band. Samples identified as positive by this method were subjected to further analysis using high-fidelity enzymes that generate products with increased accuracy for sequencing applications. Due to low *‘Ca.* L. solanacearum’ titer based upon initial PCR analyses, two different high fidelity polymerases were used for analysis of the 16S rRNA gene, 50S ribosomal proteins L10 and L12 genes, and the outer membrane protein gene. Despite repeated attempts to amplify these three genes from all specimens, for 4 of the 47 specimens, no amplicons were obtained. For the remaining specimens, all target genes were amplified and sequenced from 23 samples, a combination of any two target genes were amplified and sequenced from 9 samples, and only a single target gene was amplified from 11 samples (see Supplemental Table S2). Specific details are subsequently listed.

For 16S rRNA amplification with primers OA2 and OI2c, 20 of the 25 ‘*Ca.* L. solanacearum’-infected psyllids from 2018 and 13 of the 22 from 2019 were amplified using a 20 µl reaction consisting of 10 µl of PrimeSTAR Max Premix (2x) (Takara Bio USA, Inc., Mountain View, CA), 0.3 µl each of 20 µM stock solutions of forward and reverse primers, 2 µl 10 × Rediload (Invitrogen, Grand Island, NY), 6.4 µl H_2_O, and 1 µl nucleic acid extract. To amplify the 16S rRNA from three additional psyllid specimens from 2018, a 20 µl reaction consisting of 2 µl 10 × Advantage 2 PCR buffer (Takara Bio USA, Inc.), 0.5 µl of a 10 mM dNTP (each) mix, 0.5 µl each of 20 µM stock solutions of forward and reverse primers, 13.4 µl H_2_O, 0.1 µl Advantage 2 polymerase mix (Takara Bio USA, Inc.), 2 µl 10 × Rediload (Invitrogen), and 1 µl nucleic acid extract was used. Thermal cycling conditions for 17 of the 2018 psyllids and 7 of the 2019 psyllid specimens amplified with PrimeSTAR and the 3 psyllids amplified with Advantage 2 consisted of 94 °C for 2 min, 40 cycles of 94 °C for 30 s, 65 °C for 30 s, and 72 °C for 1 min, with a final extension of 72 °C for 5 min. Thermal cycling conditions for the remaining 2018 samples and 2019 samples were modified to a two-step program as follows: 40 cycles of 98 °C for 10 s and 68 °C for 25 s, with a final elongation of 68 °C for 5 min.

Amplification of the 50S ribosomal protein L10 and L12 genes was done using primer pair CL514F and CL514R^37^. For four of the psyllid specimens from 2018, a 20 µl PCR reaction with PrimeSTAR was used, following the conditions listed for 16S rRNA gene amplification. For eight other psyllid specimens from 2018, and eighteen of the twenty-two ‘*Ca.* L. solanacearum’-infected psyllid specimens from 2019, a 20 µl PCR reaction with Advantage 2 polymerase was used, following the conditions listed for 16S rRNA gene amplification. Thermal cycling conditions for the samples amplified with PrimeSTAR consisted of 94 °C for 3 min., 40 cycles of 94 °C for 45 s, 54 °C for 45 s, and 72 °C for 1 min, with a final extension of 72 °C for 10 min. Thermal cycling conditions for all samples amplified with Advantage 2 were modified to the following touchdown program: 94 °C for 1 min, 20 cycles of 94 °C for 15 s, 60 °C for 30 s with a touchdown decrease of 0.5 °C per cycle, and 72 °C for 45 s, 20 cycles of 94 °C for 15 s, 50 °C for 30 s, and 72 °C for 45 s, with a final elongation of 72 °C for 1 min.

Amplification of the outer membrane protein gene was done using primer pair OMB1482F and OMB2086R^38^. For five of the psyllid specimens from 2018, a 20 µl PCR reaction with PrimeSTAR was used, following the conditions listed for 16S rRNA gene amplification. For nine other psyllid specimens from 2018, and eighteen of the twenty-two ‘*Ca.* L. solanacearum’-infected psyllid specimens from 2019, a 20 µl PCR reaction with Advantage 2 polymerase was used following the conditions listed for 16S rRNA gene amplification. Thermal cycling conditions for the samples amplified with PrimeSTAR consisted of 94 °C for 2 min, 40 cycles of 94 °C for 15 s, 55 °C for 90 s, 72 °C for 60 s, with a final extension of 72 °C for 5 min. Thermal cycling conditions for samples amplified with Advantage 2 were modified to the following touchdown program: 94 °C for 1 min, 20 cycles of 94 °C for 15 s, 64 °C for 30 s with a touchdown decrease of 0.5 °C per cycle, and 72 °C for 45 s, 20 cycles of 94 °C for 15 s, 54 °C for 30 s, and 72 °C for 45 s, with a final elongation of 72 °C for 1 min.

To identify the psyllid specimens infected with ‘*Ca.* L. solanacearum,’ universal primers HCO2198 and LCO1490 that target the psyllid mitochondrial cytochrome oxidase 1 (CO1) gene^39^ were used along with a high-fidelity enzyme. A 20 µl reaction was prepared using 10 µl of PrimeSTAR Max Premix (2x) (Takara Bio USA, Inc., Mountain View, CA), 0.3 µl forward and reverse primers, 2 µL 10 × Rediload (Invitrogen, Grand Island, NY), 6.4 µl H_2_O, and 1 µl nucleic acid extract. For 22 of the 25 ‘*Ca.* L. solanacearum’-infected psyllid samples from 2018, thermal cycling conditions consisted of 95 °C for 1 min, 40 cycles of 95 °C for 15 s, 48 °C for 1 min, and 72 °C for 30 s, followed by an extension of 72 °C for 7 min. For the remaining three infected psyllids from 2018, and for 21 ‘*Ca.* L. solanacearum’-infected psyllids from 2019, modified thermal cycler conditions were used: 40 cycles of 98 °C for 10 s, 48 °C for 10 s, and 72 °C for 15 s, following by an extension of 72 °C for 5 min. To determine the taxonomic composition of the ‘*Ca.* L. solanacearum’-negative psyllids, the CO1 gene was amplified from 263 specimens selected randomly, and from 46 specimens selected because taxonomic notes were taken for the specimen prior to extraction.

All PCR products for the 16S rRNA, 50S ribosomal proteins L10 and L12, outer membrane protein, and CO1 genes were visualized on 1.5% agarose gels with ethidium bromide staining. Product sizes for each amplicon were 1168 bp, 673 bp, 605 bp, and 709 bp, respectively.

### Cloning and sequencing analysis

All PCR products were purified prior to use in cloning reactions and sequencing analysis. For 16S rRNA products, purification occurred one of three ways; PCRs were purified using either the DNA Clean and Concentrator Kits (Zymo Research, Irvine, CA) (n = 4) or the GeneJET PCR Purification Kit (Thermo Scientific, Waltham, MA) (n = 29), or bands were cut from the agarose gels and purified using GenElute minus Ethidium Bromide Spin Columns (Sigma Aldrech, Burlington, MA) (n = 3). For the 50S ribosomal subunit proteins L10 and L12 gene product, purification was done one of two ways; PCRs were purified using either the DNA Clean and Concentrator Kit (n = 2) or the GeneJET PCR Purification Kit (n = 28). For the outer membrane protein gene products, purification occurred one of three ways; PCRs were purified using either the DNA Clean and Concentrator Kits (n = 2) or the GeneJET PCR Purification Kit (n = 29), or a band was cut from the agarose gel and purified using a GenElute minus Ethidium Bromide Spin Column (n = 1). For the ‘*Ca.* L. solanacearum’-infected psyllids, CO1 products were purified one of two ways; PCRs were purified using either the DNA Clean and Concentrator Kit (n = 4) or the GeneJET PCR Purification Kit (n = 42). The CO1 products from all ‘*Ca.* L. solanacearum’-negative specimens were purified using the GeneJET PCR Purification Kit.

Of the ‘*Ca.* L. solanacearum’-infected 2018 samples, purified PCR products of the 16S rRNA, 50S ribosomal subunit, outer membrane protein, and CO1 genes were subjected to cloning and sequencing analysis for nine, five, six, and ten psyllid specimens, respectively. Of the ‘*Ca.* L. solanacearum’-infected 2019 samples, purified PCR products of the 16S rRNA, 50S ribosomal subunit, outer membrane protein, and CO1 genes from two psyllid specimens each were subjected to cloning and sequencing analysis. Briefly, an A overhang was added to the PCR product using the following reaction: 1 µl dATP, 2 µl Titanium Taq Buffer (Takara Bio USA, Inc., Mountain View, CA), 1 µl Titanium Taq, 100 ng purified PCR, and H_2_O to 20 µl total reaction volume. Thermal cycler conditions for A addition were 94 °C for 3 min and 72 °C for 20 min. All samples were then cloned into Top 10 chemically competent cells using the TOPO TA cloning kit with the pCR 2.1-TOPO vector (Invitrogen, Grand Island, NY). All plasmids were isolated from the Top 10 cells using the GeneJET Plasmid Miniprep Kit (Thermo Scientific, Waltham, MA). At least three plasmids per cloning reaction were subjected to DNA sequencing analysis at Molecular Cloning Laboratories (MCLAB; South San Francisco, CA), except for a single 2018 sample where only one clone of the 16S rRNA product was generated for sequencing. Consensus sequences were generated from the clones as described in^2^ and deposited in the National Center for Biotechnology Information (NCBI) GenBank. The nucleotide identity of each consensus sequence was assessed using the NCBI blastn suite (https://blast.ncbi.nlm.nih.gov/Blast.cgi).

All remaining purified PCR products for the four gene targets were subjected to direct sequencing using the respective forward and reverse primers. All direct sequencing was done at Molecular Cloning Laboratories. Product sizes ranged from 1027–1076 base pairs for the 16S rRNA gene, 627–637 base pairs for the 50S ribosomal subunit genes, 566–586 base pairs for the outer membrane protein genes, and 676–699 base pairs for the CO1 gene. Sequencing results were unable to be generated from any of the three ‘*Ca.* L. solanacearum’ genes targeted for one psyllid specimen from 2018 and for three psyllid specimens from 2019. For all other specimens, at least one of the targeted genes was successfully sequenced to enable identification of the ‘*Ca.* L. solanacearum’ haplotype.

The CO1 gene sequences of 46 of the 47 Klamath Basin ‘*Ca.* L. solanacearum’-positive specimens were of high quality. For a single psyllid collected in 2019, sequencing results of the CO1 gene were not clean, indicating that more than one species may have been present in the nucleic acid extraction. This contamination likely occurred while removing the specimen from the yellow sticky card. BLAST analyses were conducted for all consensus sequences using the NCBI online blastn suite (https://blast.ncbi.nlm.nih.gov/Blast.cgi). Presently, CO1 sequence data in the NCBI database is limited to Old World Aphalara species; there are no GenBank entries for New World Aphalara species, so BLAST analyses were limited.

### Identification of supplemental psyllid species

The 2,751 specimens of psyllids assayed for ‘Ca. L. solanacearum’ were collected using sticky cards. This method complicated use of standard taxonomic tools to identify species, as important morphological traits often were buried in adhesive or were deformed beyond use. This problem was magnified due to the large number of specimens belonging to the difficult-to-identify genus *Aphalara* (Aphalaridae). The large volume of trapped specimens and difficulties caused by the trapping method made it problematic to use morphology in assigning species names to trapped specimens. Instead, the CO1 gene from each *‘Ca.* L. solanacearum’-positive specimen was sequenced. Sequences were used to group specimens into genetically identical subgroups. The molecular work was then supplemented with morphological examination of additional *Aphalara* specimens collected with sweep nets or aspirators from the study region in southern Oregon and from other sites in the Pacific Northwest (Supplemental Table S1).

Morphological traits of supplementary specimens were compared with descriptions of named species^22,24,29,30,40–43^. We included comparisons of specimens with literature descriptions of Old World *Aphalara* if our specimens failed to obviously match named Nearctic species^24,44,45^. Characters of importance in separating *Aphalara* include structures associated with the head, wing markings, pattern of the spinules on surface of forewing, characteristics of the male genitalia, and structures associated with the female terminalia^22^. The abdominal terminalia of females were removed, placed in a drop of glycerol, and observed in lateral and dorsal views under a dissecting microscope. The proctiger was then removed, cleared in 10% KOH, and spread dorsal side up on a microscope slide to examine shape of the anal opening and arrangement of circumanal pores. In male specimens, the genital segment was removed, cleared in 10% KOH, and then positioned and examined in a drop of glycerol. The parameres were detached from the genital segment and placed in a drop of glycerol in lateral or dorso-lateral view. The aedeagus was positioned in lateral view under a cover slip for viewing. Forewings were examined for arrangement of spinules. Initial dissections were done under a Zeiss Stemi 2000C dissecting microscope. Dissected parts were then examined and photographed using a Leica MZ6 dissecting microscope or Leica DMLS compound microscope. The imaging device was a Luminera Infinity 2 ℓ Camera with Image-Pro 10 software by Media Cybernetics.

The new specimens were found to include a mix of seven morphospecies. Identification of these morphospecies as formally named species was of uneven certainty across the seven putative taxa, therefore we refer to specimens as *Aphalara* sp. 1, *Aphalara* sp. 2, and so forth, ending with *Aphalara* sp. 7 (Supplemental Table S1). The CO1 genes of the specimens were then direct-sequenced (as previously described) to verify that the seven species defined by morphology were also defined molecularly, and to look for genetic match of our morphospecies with sequences from the ‘*Ca.* L. solanacearum’-positive specimens that had been collected with sticky cards. Physical vouchers of morphospecies are housed at the USDA-ARS laboratory in Wapato (collection of TML and DRH) with the labels *Aphalara* sp. 1 to *Aphalara* sp. 7. Sequence data were entered into GenBank using this same nomenclature.

### Phylogenetic analysis

Phylogenetic analyses of the *‘Ca.* L. solanacearum’ 16S rRNA, 50S ribosomal protein and outer membrane protein were performed in Geneious Prime 2020.1.2 (www.geneious.com)^46^ (Supplemental Table S3). All sequence alignments were performed using the Geneious Alignment tool using default global alignment with free end gaps setting, with a gap open penalty of 15 and a gap extension penalty of 6.66. Phylogenetic trees for each target gene were constructed using RAxML (Randomized Axelerated Maximum Likelihood plugin version 4.0)^47^ in Geneious. The GTR Gamma nucleotide model with the rapid hill algorithm with 1,000 bootstraps was used as in^2^.

A similar multiple sequence alignment was done using the Geneious Alignment tool in Geneious Prime 2021.1.1^46^ for the CO1 consensus sequences generated from the ‘*Ca.* L. solanacearum’-infected specimens, supplemental specimens collected in the Northwest U.S., the Old World *Aphalara* species, the Old World, New World, and Holarctic species of *Craspedolepta,* the Old World introduction *Heterotrioza chenopodii*, and the potato psyllid, *Bactericera cockerelli* (Supplemental Table S4). No GenBank sequences were available for New World species of *Aphalara*. The top BLAST hits, Aphalarinae and unnamed species of *Craspedolepta* were also included. The *Craspedolepta* sequences were included because of their closeness to *Aphalara* and because other studies have indicated that *Craspedolepta* is a host of *‘Ca.* L. solanacearum’^20^. A phylogenetic tree was constructed using RAxML, with the GTR Gamma nucleotide model with the rapid hill algorithm with 1,000 bootstraps.

### Amino acid sequence alignment

Amino acid sequences were compared for the 50S ribosomal subunit L10 protein, the 50S ribosomal subunit L12 protein, and the outer membrane protein. All sequences listed above for the phylogenetic analysis were translated in Geneious Prime using the correct frame. Sequences consisted of 71, 104, and 191 amino acids for the L10, L12, and outer membrane proteins, respectively. All amino acid sequence alignments were performed using the Geneious Alignment tool using default global alignment with free end gaps setting, with a gap open penalty of 12 and a gap extension penalty of 3, and the percent identity matrix was generated.

## Supplementary Information

Supplementary Information.

## Data Availability

The data used and produced in this manuscript is available upon reasonable request from author Kylie Swisher Grimm. Sequence files for accessions ON569096-ON569124 are available in the NCBI GenBank database.
