# Supplementary material for: Identification of three new ‘Candidatus Liberibacter solanacearum’ haplotypes in four psyllid species (Hemiptera: Psylloidea)
Source: Sci Rep. 2022 Nov 30;12:20618. doi: 10.1038/s41598-022-24032-9 (PMC9712394; doi:10.1038/s41598-022-24032-9)
Supplement: Supplementary file 1 — Supplementary Information. [file 41598_2022_24032_MOESM1_ESM.docx]

Table S1. Collecting information and tentative identifications of supplementary specimens of *Aphalara*.

*Aphalara* sp. 1

- Specimens used in tree (Fig. 4; GenBank Accession ON569123) were collected from *Thuja* sp. windbreaks, autumn 2019, Yakima County, Washington (collectors T. Lewis and D. Horton)
- Named species providing closest match to specimens: *Aphalara loca* Caldwell, 1937.
  - Specimens match Caldwell’s 1937 description of *A. loca*, except for slight disagreement in shape of the cubital cell.
  - *Aphalara loca* is widespread in North America including the Pacific Northwest. The host of *A. loca* is listed as *Polygonum* *erectum* in [2] and [3]; no details are provided about source of host data. *Polygonum erectum* appears not to be present in the region that provided our specimens.
- Notes on *Aphalara* sp. 1
  - Likely *Aphalara loca*.
  - Host plant: we regularly collect adults and immatures on the Old World *Polygonum aviculare* (Polygonaceae), a common weed in North America.
  - This is a very common psyllid in the Pacific Northwest and is routinely collected from conifers in autumn and winter.

*Aphalara* sp. 2

- Specimens used in tree (Fig. 4; GenBank Accession ON569118) were collected from unidentified *Polygonum* (Polygonaceae), June and September 2019, Miller Island, Klamath County, Oregon (collector A. Jensen).
- Named species providing closest match to specimens: *Aphalara persicaria* Caldwell, 1937.
  - Characters of the wing and genitalia match descriptions for *A. persicaria* in [1, 4, 5, 6]. The female terminalia in our specimens differ a bit from published descriptions of *A. persicaria* in having the apex of the dorsal plate directed dorsad and by somewhat less extensive caudal expansion of the circumanal ring. The forewing in some specimens has brown suffusion on both sides of each vein, rather than having an unpatterned forewing.
  - Known distribution of *A. persicaria* is Cuba, Eastern Mexico, Eastern and Midwestern U.S. [6]. [6] list *Persicaria* (Polygonaceae) species as hosts.
- Notes on *Aphalara* sp. 2
  - Possibly an undescribed species (near *A. persicaria*). Geographic source of our specimens (southern Oregon) is well outside of the known range of *A. persicaria*.
  - Host plant in collecting region is unknown. *Persicaria lapathifolia*, a host plant of *A. persicaria* [6], is present in the collecting area.

*Aphalara* sp. 3

- Specimens used in tree (Fig. 4; GenBank Accession ON569124) were collected from unidentified *Polygonum* (Polygonaceae) and mixtures of grass herbaceous vegetation that included *Rumex* and *Polygonum* species (Polygonaceae), June and September 2019, Miller Island, Klamath County, Oregon (collector A. Jensen).
- Named species providing closest match to specimens: *Aphalara curta* Caldwell, 1937.
  - Characters of female terminalia agree well with descriptions in [1].
  - Known distribution of *A. curta* includes midwestern and western North America [2]. Host plant is unknown [2].
- Notes on *Aphalara* sp. 3
  - Tentatively recognized here as *Aphalara curta*.
  - Host plant in collecting region is unknown

*Aphalara* sp. 4

- The specimen used in tree (Fig. 4; GenBank Accession ON569119) was collected from unidentified *Rumex* (Polygonaceae), July 2020, Lake County, Oregon (collector A. Jensen).
- Named species providing closest match to specimen: *Aphalara rumicis* Mally, 1894.
  - The specimen is placed in the *rumicis* species group of [7] in having the following combination of traits: short and blunt exposed clypeus; bluntly angulate apex of paramere; aedeagus with straight pointed apico-ventral hook and concave apico-ventral process; and circumanal ring of female having two rows of pores. However, the aedeagus and the tooth on the paramere differ somewhat from the illustrations of *A. rumicis* in [8] and [7].
  - The known distribution of *A. rumicis* is midwestern and western North America with records from both Oregon and Washington [2]. The host plant is *Rumex altissimus* [2], which does not occur in western North America.
- Notes on *Aphalara* sp. 4
  - From traits of the male genitalia this possibly is *A. rumicis*, or it is an undescribed species near *A. rumicis*.
  - Host plant is unknown.

*Aphalara* sp. 5

- Specimens used in tree (Fig. 4; GenBank Accession ON569120) were collected from unidentified *Polygonum* (Polygonaceae) and from a mixture of grass/herbaceous vegetation that included *Rumex* and *Polygonum* species (Polygonaceae), June and September 2019, Miller Island, Klamath County, Oregon (collector A. Jensen).
- Named species providing closest match to specimens: *Aphalara nubifera* Patch, 1912.
  - Specimens were compared to descriptions of *A. nubifera* in [1, 6, 8]. The paramere of the male genitalia and distribution of spinules on forewings matched descriptions well. The female terminalia differ from descriptions in that the circumanal pore ring has two rows, widening to four rows in the caudal third, instead of 3-4 rows throughout in published descriptions. The markings of the forewings differ from descriptions in lacking a cloudy transverse band.
  - Known distribution of *A. nubifera* is western North America [2]. The host plant (from Ft. Collins, Colorado records in [8] is a species of Brassicaceae (*Descurainia pinnata*) rather than Polygonaceae.
- Notes on *Aphalara* sp. 5
  - Very tentatively recognized here as *Aphalara nubifera.*
  - The host plant in the collecting region is unknown. *Sisymbrium canescens* (syn. of *Descurainia pinnata*), listed as the host of *A. nubifera* by [2], is widespread in the Pacific Northwest, and it is conceivable that it was present but unnoticed at the collecting site in Klamath County, Oregon.

*Aphalara* sp. 6

- Specimens used in tree (Fig. 4; GenBank Accession ON569121) were collected from unidentified *Polygonum* (Polygonaceae) “in dry ditch”, June 2019 Miller Island, Klamath County, Oregon (collector A. Jensen); and from *Barbarea orthoceras* (Brassicaceae), Fremont National Forest, Lake County, Oregon, July 2020 (collector A. Jensen).
- Named species providing closest match to specimens: *Aphalara maculata* Caldwell, 1937.
  - Forewing (maculation) patterns in specimens agree with description of *A. maculata* in [1]. Male genitalia of specimens depart somewhat from illustrations in [7]: paramere with a more flattened apex and with the thumb-like subapical anterior process placed higher; the beak of the apical dilation of the aedeagus is directed more nearly perpendicular to the longitudinal axis of the segment.
  - *Aphalara maculata* has been reported only from California [2]. Host plant is unknown.
- Notes on *Aphalara* sp. 6
  - Tentatively identified as *Aphalara maculata*. The contradictions in genitalia between our specimens and illustrations in the literature conceivably are within the range of normal variation.
  - Host plant: The developmental hosts are Brassicaceae rather than Polygonaceae. We have collected adults and immatures of this species in Washington and Montana from *Rorippa palustris* (L.) Besse, *Barbarea orthoceras* Ledeb., and *B. vulgaris* W.T. Aiton.

*Aphalara* sp. 7

- Specimens used in tree (Fig. 4; GenBank Accession ON569122) were collected from *Rumex occidentalis* (Polygonaceae), June 2020, Fremont National Forest, Lake County, Oregon, July 2020 (collector A. Jensen); and, from *Rumex crispus* (Polygonaceae), June 2019 Miller Island, Klamath County, Oregon (collector A. Jensen).
- Named species providing closest match to specimens: *Aphalara simila* Caldwell, 1937.
  - Male parameres and female terminalia match descriptions in [1] but differ from descriptions in [5] in shape of the aedeagus, slight differences in the paramere, and in arrangement of spinules on the forewing membrane. In our specimens, the shaft of distal segment of aedeagus is not straight; the thumb-like process on the apex of the paramere is smaller than shown in illustrations; and spinules are arranged in irregular lines rather than as rhomboids or squares in some female specimens.
  - Known distribution of *A. simila* includes Mexico and the western U.S. with records from both Oregon and Washington [1, 2, 5]. Host plant is listed as unknown (Hodkinson 1988) or *Rumex* sp. [5, 7].
- Notes on *Aphalara* sp. 7
  - Uncertain identity, but possibly *Aphalara simila*.
  - Host plant in the Oregon collecting region is unknown. In Washington State we collect adults and immatures of this species from both *Rumex salicifolius* and *Rumex crispus* (Polygonaceae).

Table S2. ‘*Ca.* L. solanacearum’-infected psyllid samples collected in 2018 and 2019 determined from initial PCR analyses are listed. Subsequent amplification and sequencing success is indicated by an ‘X’ for each of the three ‘*Ca.* L. solanacearum’ target genes (16S rRNA, 50S ribosomal proteins L10/L12, and the outer membrane protein (OMB)), as well as the psyllid CO1 gene. If amplification or sequencing was poor for a specific gene, it is denoted by ‘-’, indicating the lack of sequencing data. Further detail is provided to indicate if an amplicon was cloned prior to sequencing, or if the amplicon was directly sequenced without cloning.

| **Year Collected** | **Sample Number** | **16S rRNA** | **50S ribosomal proteins** | **OMB** | **CO1** |
| --- | --- | --- | --- | --- | --- |
| 2018 | 29 | X (clones) | X (clones) | X (clones) | X (clones) |
|  | 63 | X (clones) | X (clones) | X (clones) | X (clones) |
|  | 141 | X (clones) | - | - | X (clones) |
|  | 147 | X (clones) | - | - | X (clones) |
|  | 908 | X (clones) | X (clones) | X (clones) | X (clones) |
|  | 931 | X (clones) | X (clones) | X (clones) | X (clones) |
|  | 935 | X (direct) | X (direct) | X (direct) | X (direct) |
|  | 936 | X (direct) | X (direct) | X (direct) | X (direct) |
|  | 938 | X (direct) | X (direct) | X (direct) | X (direct) |
|  | 988 | X (direct) | X (direct) | X (direct) | X (direct) |
|  | 999 | X (direct) | X (direct) | X (direct) | X (direct) |
|  | 1018 | - | - | - | X (direct) |
|  | 1035 | - | X (direct) | X (direct) | X (direct) |
|  | 1054 | X (direct) | - | - | X (direct) |
|  | 1059 | X (clones) | X (clones) | X (clones) | X (clones) |
|  | 1063 | X (direct) | - | - | X (direct) |
|  | 1076 | X (direct) | - | - | X (direct) |
|  | 1080 | X (direct) | - | - | X (direct) |
|  | 1086 | X (direct) | - | - | X (direct) |
|  | 1092 | X (direct) | - | - | X (direct) |
|  | 1109 | X (direct) | - | - | X (clones) |
|  | 1112 | X (direct) | - | - | X (direct) |
|  | 1139 | X (direct) | - | X (direct) | X (direct) |
|  | 1145 | X (clone) | - | X (direct) | X (clones) |
|  | 1148 | X (clones) | X (direct) | X (clones) | X (clones) |
| 2019 | 11 | X (direct) | - | - | X (direct) |
|  | 21 | - | X (direct) | X (direct) | X (direct) |
|  | 39 | - | X (direct) | X (direct) | X (direct) |
|  | 62 | X (direct) | X (direct) | X (direct) | X (direct) |
|  | 135 | - | - | - | X (direct) |
|  | 140 | X (direct) | X (direct) | X (direct) | X (direct) |
|  | 141 | X (direct) | X (direct) | X (direct) | X (direct) |
|  | 279 | - | X (direct) | X (direct) | X (direct) |
|  | 300 | - | - | - | X (direct) |
|  | 653 | X (clones) | X (clones) | X (clones) | X (clones) |
|  | 654 | X (direct) | X (direct) | X (direct) | X (direct) |
|  | 655 | X (direct) | X (direct) | X (direct) | X (direct) |
|  | 764 | X (direct) | X (direct) | X (direct) | X (direct) |
|  | 909 | - | X (direct) | X (direct) | X (direct) |
|  | 920 | X (direct) | X (direct) | X (direct) | X (direct) |
|  | 931 | X (direct) | X (direct) | X (direct) | X (direct) |
|  | 996 | - | X (direct) | X (direct) | X (direct) |
|  | 1015 | X (direct) | X (direct) | X (direct) | - |
|  | 1048 | X (direct) | X (direct) | X (direct) | X (direct) |
|  | 1199 | X (clones) | X (clones) | X (clones) | X (clones) |
|  | 1205 | - | - | - | X (direct) |
|  | 1214 | - | X (direct) | X (direct) | X (direct) |

Table S3. Phylogenetic analyses of the *‘Ca.* L. solanacearum’ 16S rRNA, 50S ribosomal protein and outer membrane protein (OMB) were performed using GenBank accessions listed. Lack of sequence availability is denoted by ‘-’.

| **Species and haplotype** | **16S rRNA** | **50S ribosomal proteins** | **OMB** |
| --- | --- | --- | --- |
| *Ca.* L. solanacearum Haplotype A | FJ498802 [9] | EU834131 [10] | JN848754 [11] |
| *Ca.* L. solanacearum Haplotype B | FJ829813 [12] | FJ498805 [13]  FJ498807 [14]  JF811598 [15] | KC768327 [16] |
| *Ca.* L. solanacearum Haplotype C | GU373048 [17]  KX431890 [18] | GU373051 [17] | KP760078 [19] |
| *Ca.* L. solanacearum Haplotype D | HQ454302 [20]  MG657031 [21] | HQ454317 [22]  KY777462 [23] | KY595979 [24] |
| *Ca.* L. solanacearum Haplotype E | KF737348 [25] | KY777461 [23] | - |
| *Ca.* L. solanacearum Haplotype F | MH259699 [26] | MH259700 [26] | MH259701 [26] |
| *Ca.* L. solanacearum Haplotype G | MN256493 [27]  MN256495 [27] | SRR8512499 [27] | - |
| *Ca.* L. solanacearum Haplotype H | MK800167 [28] | MK800169 [28] | - |
| *Ca.* L. solanacearum Haplotype U | MG701016 [29] | MG701023 [29] | MT238983 [30] |
| *Ca.* L. solanacearum Haplotype Cras1a | MT229445 [30] | MT249177 [30] | MT238970 [30] |
| *Ca.* L. solanacearum Haplotype Cras1b | MT229446 [30] | MT249178 [30] | MT238968 [30] |
| *Ca.* L. solanacearum Haplotype Cras2 | MT249462 [30] | MT249190 [30] | MT238975 [30] |
| *Ca.* L. africanus | KY000560 [31] | U09675 [32] | - |
| *Ca.* L. americanus | EU921623 [33] | EF122254 [34] | - |
| *Ca.* L. asiaticus | KY990822 [35] | DQ471904 [36] | AB741531 [37] |
| *Ca.* L. brunswickensis | KY077741 [38] | - | - |
| *Ca.* L. ctenarytaina | KX768754 [39] | KX810197 [39] | - |
| *Ca.* L. europaeus | JX244259 [40] | - | - |

Table S4. Phylogenetic analysis of the psyllid CO1 gene was performed using the GenBank accessions listed.

| **Psyllid sample** | **GenBank Accession Number** | **Reference** |
| --- | --- | --- |
| *Aphalara* sp. 1 | ON569108 | this study |
| *Aphalara* sp. 1 | ON569109 | this study |
| *Aphalara* sp. 2 | ON569110 | this study |
| *Aphalara* sp. 2 | ON569111 | this study |
| *Aphalara* sp. 2 | ON569112 | this study |
| *Aphalara* sp. 3 | ON569113 | this study |
| *Aphalara* sp. 3 | ON569114 | this study |
| *Aphalara* sp. 3 | ON569115 | this study |
| *Aphalara* sp. 3 | ON569116 | this study |
| *Heterotrioza* *chenopodii* | ON569117 | this study |
| *Aphalara* sp. 2 (*A. persicaria*-like) | ON569118 | this study |
| *Aphalara* sp. 4 (*A. rumicis*-like) | ON569119 | this study |
| *Aphalara* sp. 5 (*A. nubifera*-like) | ON569120 | this study |
| *Aphalara* sp. 6 (*A. maculata*-like) | ON569121 | this study |
| *Aphalara* sp. 7 (*A. simila*-like) | ON569122 | this study |
| *Aphalara* sp. 1 (*A. loca*-like) | ON569123 | this study |
| *Aphalara* sp. 3 (*A. curta*-like) | ON569124 | this study |
| *Aphalara avicularis* | MT021761 | [41] |
| *Aphalara itadori* | KP113673 | [42] |
| *Aphalara maculipennis* | KU517185 | [43] |
| *Aphalara polygoni* | MG988639 | [44] |
| *Craspedolepta alaskensis* | KU874725 | [45] |
| *Craspedolepta angustipennis* | JF884002 | iBOL project 37833 |
| *Craspedolepta anomala* | MG988707 | [44] |
| *Craspedolepta canadensis* | MG514084 | BioProject: PRJNA472144 |
| *Craspedolepta gutierreziae* | MT021786 | [41] |
| *Craspedolepta minutissima* | MT021789 | [41] |
| *Craspedolepta nebulosa* | MG988708 | [44] |
| *Craspedolepta nervosa* | MT021790 | [41] |
| *Craspedolepta subpunctata* | MT021791 | [41] |
| *Heterotrioza chenopodii* | MT021799 | [41] |
| *Bactericera cockerelli* | MT040966 | [41] |
| Aphalarinae | KR582021 | [46] |
| *Craspedolepta* sp. | KR578677 | [46] |
| *Craspedolepta* sp. | MG401317 | BioProject: PRJNA472144 |

**References Cited**

1. Caldwell, J.S. Some North American relatives of *Aphalara calthae* Linnaeus (Homoptera: Chermidae). *Annals Entomol. Soc. America* **30,** 563-569 (1937).
2. Hodkinson, I.D. The Nearctic Psylloidea (Insecta: Homoptera): an annotated check list. *J. Nat. Hist.* **22,** 1179-1243 (1988).
3. Percy, D.M., Rung, A. & Hoddle, M.S. An annotated checklist of the psyllids of California (Hemiptera: Psylloidea). *Zootaxa* **3193,** 1-27 (2012).
4. Caldwell, J.S. The jumping plant lice of Ohio (Homoptera, Chermidae). *Bull. Ohio Biological Survey* **34,** 229-281 (1938).
5. Burckhardt, D., Cort, G.D. & de Queiroz, D.L. Jumping plant lice of the genus *Aphalara* (Hemiptera, Psylloidea, Aphalaridae) in the Neotropics. *ZooKeys* **980,** 119-140 (2020).
6. Halbert, S.E. & Burckhardt, D. The psyllids (Hemiptera: Psylloidea) of Florida: newly established and rarely collected taxa and checklist. *Insecta Mundi* **0788,** 1-88 (2020).
7. Burckhardt, D. & P. Lauterer. Systematics and biology of the *Aphalara exilis* (Weber & Mohr) species assemblage (Hemiptera: Psyllidae). *Entomologica scandinavica* **28,** 271-305 (1997).
8. Patch, E.M. Notes on Psyllidae. *Maine Agricultural Experiment Station* **#55,** 215-234 (1912).
9. Crosslin, J.M. & Bester, G. First report of *Candidatus* Liberibacter psyllaurous in zebra chip symptomatic potatoes from California. *Plant Dis.* **93,** 551 (2009).
10. Liefting, L.W. et al. ‘*Candidatus* Liberibacter solanacearum’, associated with plants in the family Solanaceae. *Plant Dis.* **93,** 208-214 (2009).
11. Crosslin, J.M. et al. First report of zebra chip disease and ‘*Candidatus* Liberibacter solanacearum’ on potatoes in Oregon and Washington State. *Plant Dis.* **96,** 452 (2012).
12. Wen, A. et al. Detection, distribution, and genetic variability of *Candidatus* Liberibacter species associated with zebra complex disease of potato in North America. *Plant Dis.* **93,** 1102-1115 (2009).
13. Crosslin, J.M. & Goolsby, J.A. New species of *Candidatus* Liberibacter associated with potato zebra chip disease found in wolfberry (*Lycium berlandieri*) in Texas. (Unpublished).
14. Munyaneza, J.E., Sengoda, V.G., Crosslin, J.M., De la Rosa-Lorzano, G. & Sanchez, A. First report of *Candidatus* Liberibacter psyllaurous in potato tubers with zebra chip disease in Mexico. *Plant Dis.* **93,** 552. (2009).
15. Ling, K.-S., Lin, H., Lewis Ivey, M.L., Zhang, W. & Miller, S.A. First report of *Candidatus* Liberibacter solanacearum naturally infecting tomatoes in the State of Mexico, Mexico. *Plant Dis.* **95,** 1026 (2011).
16. Bextine, B.R. et al. First report of ‘*Candidatus* Liberibacter solanacearum’ on tomato in El Salvador. *Plant Dis.* **97,** 1245 (2013).
17. Munyaneza, J.E. et al. First report of ‘*Candidatus* Liberibacter solanacearum’ associated with psyllid-affected carrots in Europe. *Plant Dis.* **94,** 639 (2010).
18. Wang, J. et al. Genomic sequence of *‘Candidatus* Liberibacter solanacearum’ haplotype C and its comparison with haplotype A and B genomes. *PLoS ONE* **12,** e0171531 (2017).
19. Munyaneza, J.E. et al. First report of ‘*Candidatus* Liberibacter solanacearum’ associated with psyllid-infested carrots in Germany. *Plant Dis.* **99,** 1269 (2015).
20. Alfaro-Fernandez, A. et al. First report of ‘*Candidatus* Liberibacter solanacearum’ in carrot in mainland Spain. *Plant Dis.* **96,** 582 (2012).
21. Mawassi, M. et al. ‘*Candidatus* Liberibacter Solanacearum’ is tightly associated with carrot yellows symptoms in Israel and transmitted by the prevalent psyllid vector *Bactericera trigonica*. *Phytopath.* **108,** 1056-1066 (2018).
22. Alfaro-Fernandez, A., Siverio, F., Cebrian, C., Villaescusa, F.J. & Font, M.I. First report of ‘*Candidatus* Liberibacter solanacearum’ associated with *Bactericera trigonica-*affected carrots in the Canary Islands. *Plant Dis.* **96,** 581 (2012).
23. Othmen, S.B. et al. ‘*Candidatus* Liberibacter solanacearum’ haplotypes D and E in carrot plants and seeds in Tunisia. *J. Plant Path.* **100,** 197-207 (2018).
24. Holeva, M.C., Glynos, P.E. & Karafla, C.D. First report of *Candidatus* Liberibacter solanacearum on carrot in Greece. *Plant Dis.* **101,** 1819 (2017).
25. Teresani, G.R. et al. Association of ‘*Candidatus* Liberibacter solanacearum’ with a vegetative disorder of celery in Spain and development of a real-time PCR method for its detection. *Phytopath.* **104,** 804-811 (2014).
26. Swisher Grimm, K.D. & Garczynski, S.F. Identification of a new haplotype of ‘*Candidatus* Liberibacter solanacearum’ in *Solanum tuberosum*. *Plant Dis.* **103,** 468-474 (2019).
27. Mauck, K.E., Sun, P., Meduri, V. & Hansen, A.K. New *Candidatus* Liberibacter psyllaurous haplotype resurrected from a 49-year-old specimen of *Solanum umbelliferum*: a native host of the psyllid vector. *Sci. Rep.* **9,** 9530 (2019).
28. Haapalainen, M. et al. A novel haplotype of ‘*Candidatus* Liberibacter solanacearum’ found in Apiaceae and Polygonaceae family plants. *Eur. J. Plant Pathol.* **156,** 413-423 (2020).
29. Haapalainen, M. et al. Genetic variation of ‘*Candidatus* Liberibacter solanacearum’ haplotype C and identification of a novel haplotype from *Trioza urticae* and stinging nettle. *Phytopath.* **108,** 925-934 (2018).
30. Sumner-Kalkun, J. et al. ‘*Candidatus* Liberibacter solanacearum’ distribution and diversity in Scotland and the characterisation of novel haplotypes from *Craspedolepta* spp. (Psyllidae: Aphalaridae). *Sci. Rep.* **10 (16567),** 1-11 (2020).
31. Roberts, R. & Pietersen, G. A novel subspecies of ‘*Candidatus* Liberibacter africanus’ found on native *Teclea gerradii* (Family: Rutaceae) from South Africa. *Antonie Van Leeuwenhoek* **110(3),** 437-444 (2017).
32. Planet, P., Jagoueix, S., Bove, J.M. & Garnier, M. Detection and characterization of the African citrus greening liberobacter by amplification, cloning, and sequencing of the rplKAJL-rpoBC operon. *Curr. Microbiol.* **30(3),** 137-141 (1995).
33. Lin, H. et al. Molecular characterization and phylogenetic analysis of 16S rRNA from a new ‘*Candidatus* Liberibacter’ strain associated with zebra chip disease of potato (*Solanum tuberosum* L.) and the potato psyllid (*Bactericera cockerelli* Sulc). *J. Plant Pathol.* **91,** 215-219 (2009).
34. Teixeira, D.C. et al. The tufB-secE-nusG-rplKAJL-rpoB gene cluster of the liberibacters: sequence comparisons, phylogeny and speciation. *Int. J. Syst. Evol. Microbiol.* **58,** 1414-1421 (2008).
35. Alizadeh, H. et al. First report of a new citrus decline disease (CDD) in association with double and single infection by *Candidatus* Liberibacter asiaticus and *Candidatus* Phytoplasma aurantifolia related strains in Iran. *Plant Dis.* **101,** 2145 (2017).
36. Teixeira, D., Eveillard, S., Martins, E., Ayres, J. & Bove, J. *Candidatus* Liberibacter asiaticus associated with citrus Huanglongbing (greening disease) in Brazil. (Unpublished).
37. Ukuda-Hosokawa, R. et al. Infection density dynamics of the citrus greening bacterium ‘*Candidatus* Liberibacter asiaticus’ in field populations of the psyllid *Diaphorina citri* and its relevance to the efficiency of pathogen transmission to citrus plants. *Appl. Environ. Microbiol.* **81,** 3728-3736 (2015).
38. Morris, J. M. et al. Novel ‘*Candidatus* Liberibacter’ species identified in the Australian eggplant psyllid, *Acizzia solanicola.* *Microb. Biotechnol.* **10,** 833-844 (2017).
39. Thompson, S.M. & Smith, G.R. ‘*Candidatus* Liberibacter fuchsiae’ associated with the New Zealand native fuchsia psyllid, *Ctenarytaina fuchsiae*. (Unpublished).
40. Thompson, S.M. et al. First report of ‘*Candidatus* Liberibacter europaeus’ associated with psyllid infested Scotch broom. *New Dis. Rep.* **27,** 6 (2013).
41. Sumner-Kalkun, J.C. et al. A diagnostic real-time PCR assay for the rapid identification of the tomato-potato psyllid, *Bactericera cockerelli* (Šulc, 1090) and development of a psyllid barcoding database. *PLoS ONE* **15(3),** e0230741 (2020).
42. Andersen, J.C., Bourchier, R.S., Grevstad, F.S., Van Driesche, R. & Mills, N.J. Development and verification of SNP arrays to monitor hybridization between two host-associated strains of knotweed psyllid, *Aphalara itadori.* *Biol. Control* **93,** 49-55 (2016).
43. Hodgetts, J. et al. DNA barcoding for biosecurity: case studies from the UK plant protection program. *Genome* **59,** 1033-1048 (2016).
44. Percy, D.M. et al. Resolving the psyllid tree of life: phylogenomic analyses of the superfamily Psylloidea (Hemiptera). *System. Entomol.* **43,** 762-776 (2018).
45. Sikes, D.S. et al. Building a DNA barcode library of Alaska’s non-marine arthropods. *Genome* **60,** 248-259 (2016).
46. Herbert, P.D.N. et al. Counting animal species with DNA barcodes: Canadian insects. *Philos. Trans. Royal Soc. B* **371,** 20150333 (2016).
